# Pubertal dysfunctions in intracranial germ cell tumors

**DOI:** 10.3389/fendo.2026.1736152

**Published:** 2026-05-19

**Authors:** Cristina Partenope, Sabrina Criscuolo, Fernando Carceller, Assunta Albanese

**Affiliations:** 1 Division of Pediatrics, Department of Health Science University of Piemonte Orientale, Ospedale Maggiore della Carità, Novara, Italy; 2 Department of Paediatric Endocrinology, Royal Marsden NHS Foundation Trust, London, United Kingdom; 3 Pediatric Unit, Department of Maternal and Child Health, University Hospital “San Giovanni di Dio e Ruggi d’Aragona”, Salerno, Italy; 4 Paediatric and Adolescent Neuro-Oncology and Drug Development Team, Children and Young People’s Unit, Royal Marsden Hospital and Institute of Cancer Research, Sutton, United Kingdom

**Keywords:** adolescents, germ cell tumor, hypogonadotrophic hypogonadism, pediatrics, precocious puberty, suprasellar tumor

## Abstract

**Introduction:**

Pediatric intracranial germ cell tumors (IC-GCTs) are frequently associated with pubertal dysfunctions, either at diagnosis or during follow-up. This study aims to evaluate the prevalence and types of pubertal disorders in a cohort of pediatric patients with IC-GCTs.

**Methods:**

We collected clinical, radiological, histopathological, and hormonal data. Pubertal dysfunctions were classified as: (1) pseudo-precocious puberty (PPP, gonadotrophin-independent); (2) central precocious puberty (CPP, gonadotrophin-dependent); (3) hypogonadotrophic hypogonadism (HH).

**Results:**

Sixty patients with IC-GCTs (followed-up between 1996 and 2023 at Royal Marsden Hospital, UK) were included (median follow-up 22.9 months). Germinomas represented 70%, teratomas and mixed GCTs 15% each. Tumors were suprasellar (59%), pineal (30%), or bifocal (11%). Treatments included surgery (11%), chemotherapy (78%), and radiotherapy (90%). Pubertal disorders were found in 27 patients (16 males, 11 females). PPP was found in five patients at diagnosis; in three, it resolved with tumor treatment; one required treatment with bicalutamide/anastrozole and one progressed to CPP. Three patients in total developed CPP (one after PPP, one post-PPP with intervening treatment, and one post-cancer treatment completion). HH was diagnosed in 20 cases (mean age 17.5 years in males, 15.1 in females). Two had HH at diagnosis. One female had amenorrhea with polycystic ovary syndrome and responded to combined oral hormonal therapy. No cases of hypergonadotrophic hypogonadism from chemotherapy were observed.

**Conclusion:**

Pubertal dysfunctions are common in IC-GCTs, especially with suprasellar involvement. Patterns include PPP, CPP, and HH, which may evolve over time. Long-term specialist endocrine follow-up is essential.

## Introduction

Brain tumors are the most frequent type of solid tumors in children and adolescents and the most common cause of pediatric oncological morbidity and mortality (1). Intracranial germ cell tumors (IC-GCTs) account for approximately 3% of them, with higher incidence in Japan (15.3% of pediatric brain tumors) (2), male sex and during the second decade of life (3). According to the latest World Health Organization classification system, IC-GCTs include germinomas and a variety of histologically distinct tumors collectively known as non-germinomatous GCTs (NGGCTs) (4). The distinction between germinomas and NGGCTs is critical since the former have a more favorable prognosis and require less intensive therapy. Tumor markers, such as alpha-fetoprotein (AFP) and β-human chorionic gonadotropin (HCG), are helpful in making this distinction, although histologic examination is often required for a definitive diagnosis. Highly elevated levels of HCG or AFP can be diagnostic of a NGGCT, whereas AFP is not elevated in germinomas and a mild elevation in beta-HCG can be seen in some HCG secreting germinomas (1, 5).

IC-GCTs arise almost exclusively from midline locations. The two most frequent sites are the pineal gland and the suprasellar region. At diagnosis suprasellar GCTs most commonly present with hypothalamic/pituitary dysfunctions, sometimes preceding the onset of visual defects or neurological signs (6, 7). The endocrinopathy often persists (or arise ex novo) after treatment due to side effects to radiotherapy/chemotherapy and surgery, as for other brain tumors (8). With particular focus on pubertal disorders, adolescents with IC-GCT may show delayed or arrested puberty, whereas precocious puberty (PP) is more common in younger children (<1% vs 14%) (9). PP can be either gonadotrophin-dependent (central-PP) or gonadotrophin-independent (Pseudo-PP) when the tumor secretes beta-HCG which directly stimulates gonadal maturation. Moreover, high dose chemotherapy with alkylating agents is associated with gonadal damage leading to hypergonadotrophic hypogonadism. In a previous bicentric study, pubertal dysfunctions were present in the 49.5% of the cohort (7). In this single-center case series, we will outline the pubertal disorders associated with IC-GCTs at diagnosis or as a consequence of the treatments in a cohort of pediatric patients.

## Materials and methods

### Patients

A retrospective analysis was conducted on data from pediatric patients (<18 years old) who were diagnosed with IC-GCT between January 1996 and March 2023 at the Royal Marsden Hospital in Sutton, UK. Patients underwent axial computed tomography or magnetic resonance imaging (MRI), and diagnosis was confirmed by histology when needed. We recorded patient demographics, measurements of height and weight, pubertal status at diagnosis and during follow-up, timing and features of symptoms at diagnosis, laboratory data such as tumor markers, hormonal levels and tumor characteristics (location, histology, and staging). The diagnosis of pubertal dysfunctions was derived from the patient’s medical history, physical examination (Tanner stage), and hormonal tests including gonadotrophins [luteinizing hormone (LH) and follicle-stimulating hormone (FSH)], sex hormones (estradiol and testosterone), thyroid hormones, and adrenal hormones. Pubertal dysfunctions were classified as:

1. Pseudo-precocious puberty (PPP) is a condition that involves the early development of secondary sexual characteristics, without the activation of the hypothalamic-pituitary-gonadal axis. PPP diagnosis was supported by low/prepubertal gonadotrophin levels and elevated sex steroids
2. Central precocious puberty (CPP), also known as gonadotropin-releasing hormone (GnRH)-dependent precocious puberty, is a medical condition that occurs when the hypothalamic-pituitary-gonadal axis is activated prematurely, leading to the early onset of secondary sexual characteristics
3. Hypogonadotropic hypogonadism (HH) is a medical condition that results in insufficient secretion of GnRH from the hypothalamus and/or reduced release of gonadotrophins (LH and FSH) by the pituitary gland. This can result in reduced or absent production of sex hormones such as testosterone in males and estrogen in females, which in turn may lead to impaired sexual development.

### Statistical analysis

Continuous variables are presented as medians and interquartile ranges (IQR), whereas categorical variables are presented as numbers/percentages. Statistical analyses were performed using SPSS Statistics (v.24; IBM Corp.-USA) and RStudio (v.1.3.1093; RStudio, PBC).

## Results

The study included 60 patients (44 males and 16 females) with a median follow-up of 22.9 months (IQR 17.7-114.4); 19/60 were followed-up for less than 3 years since tumor diagnosis.

**Figure 1:**
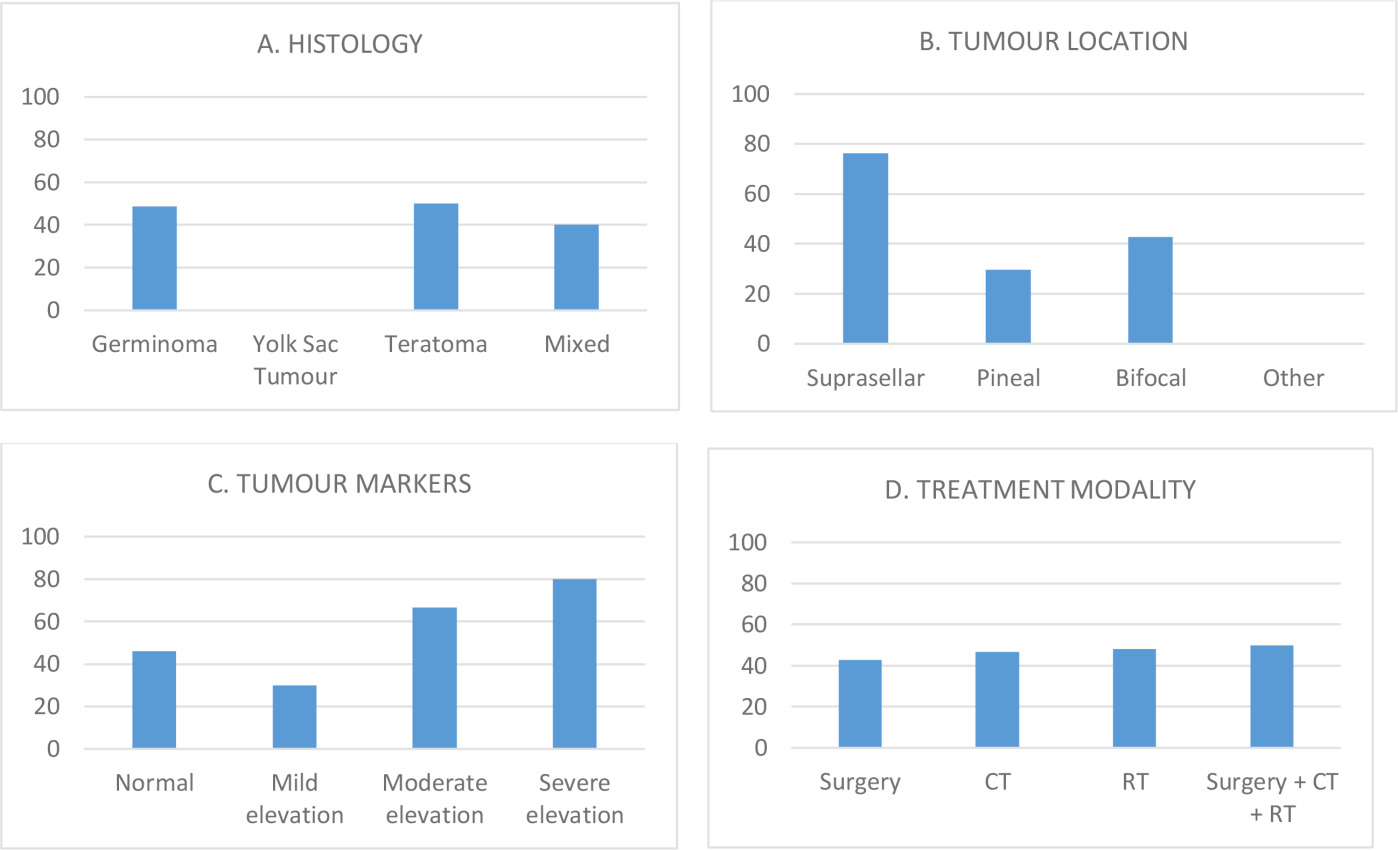
Prevalence (%) of pubertal dysfunction in the cohort stratified by tumor location **(A)**, histological subtype **(B)**, tumor marker profile **(C)**, or treatment modality **(D)**.Serum tumor markers were classified as follows: normal (HCG < 5 U/L), mildly (HCG 5–25 U/L), moderately (HCG 25–500 U/L), and severely (HCG > 500 U/L) elevated.

The median age at tumor diagnosis was 12 (IQR 1–18) years, with a peak age at diagnosis between 9 and 10 years. Adolescents aged >11 years accounted for 60% of cases. Histologically, 70% had germinomas, 15% mature teratomas, 15% mixed GCT. IC-GCTs were suprasellar (59%), pineal (30%) or bifocal (11%). A surgical procedure for biopsy or gross tumor resection was performed in 47 patients (78%); treatment included surgery (11%), chemotherapy (78%) and radiotherapy (RT) (90%). Radiation therapy was delivered to 54 out of 60 patients (90%), with a median age of 11.6 years (range 5.4–18.3) at first radiation treatment. Among them, 19/60 patients (32%) received craniospinal irradiation of 24–30.6 Gy (with boosted metastasis irradiation of 16 Gy in 12 cases), 9/60 patients (15%) whole brain irradiation of 54 Gy, 2/60 patients (3%) whole ventricular field irradiation (WVI) of 24 Gy, 19/60 patients (32%) WVI of 24 Gy with primary site simultaneous integrated boost (16 Gy) and 5/60 patients (8%) received focal irradiation only (40–54 Gy). Most patients (29/60: 48%) were prepubertal at the time of diagnosis. A total of 27 subjects (59% males) exhibited a pubertal disorder (**Table 1**; **Supplementary Table 1**). Among them, 16 presented with a pre-pubertal Tanner stage, while 11 were classified as post-pubertal. **Figure 1** shows the prevalence of pubertal dysfunction in the cohort stratified for histology, tumor location, treatment modality and tumor markers.

**Table 1:** Patients displaying pubertal dysfunctions.

Pubertal dysfunction	Tumor histology and location	Age, sex and timing	Treatment
20 HH (10 M, 10 F) arrested or delayed puberty	15 germinoma, 2 teratoma, 3 mixed histology 14 suprasellar, 3 pineal, 3 bifocal	Mean age 17.5 years for males and 15.1 for females 28.2 months (IQR 17.7-50.8) after end of oncologic treatment, 2 cases before tumor diagnosis	EE+progesteron Testosterone
1 secondary amenorrhea hypothalamic dysfunction +PCOS	Suprasellar germinoma	19.2-year-old female 35.1 months after end of oncologic treatment	EE+progesteron
5 PPP (5 M)		At IC-GCT diagnosis	
	Pineal germinoma	6.8-year-old boy developed CPP soon after diagnosis	Agonist analogue of GnRH
	Mixed histology, pineal	6.8-year-old boy developed CPP after 14 months	Bicalutamide and anastrozole for 14 months → agonist analogue of GnRH
	2 pineal germinoma 1 suprasellar teratoma	3 male subjects (aged 9.1,10.8 and 10.6, respectively) showed spontaneous resolution with oncological therapy and concomitant ↓ HCG levels - one of them was then diagnosed with HH at 13.4 years of age	No therapy Testosterone
1 CPP (1 M)	Pineal germinoma	9.8-year-old male 3.7 months after end of oncologic treatment	Agonist analogue of GnRH
No cases of hypergonadotrophic hypogonadism due to chemotherapy-related gonadal toxicity			

HH, Hypogonadotrophic hypogonadism; M, male; F, female; EE, etinilestradiol; PCOS, polycystic ovary syndrome; PPP, Pseudo-precocious puberty; IC-GCT, intracranial germ cell tumors; CPP, Central precocious puberty; GnRH, gonadotropin-releasing hormone.

PPP was evident in 5 patients at diagnosis, all males with HCG secreting-GCTs: in three patients it reverted spontaneously accordingly with tumor cure and concomitant reduction of HCG levels; one of them was diagnosed with HH 5.3 years later and was then treated with testosterone. One patient with PPP subsequently developed CPP and received therapy with an agonist analogue of gonadotropin-releasing hormone for 5 years; one more subject was initially treated with bicalutamide (androgen receptor blocker) and anastrozole (aromatase inhibitor) daily for 14 months and then with triptorelin after he developed CPP (still ongoing at the time of writing). Moreover, only one male subject manifested CPP 3.7 months after the completion of oncologic therapies and received treatment with triptorelin. Low gonadotrophin levels were found in 20 cases (33%; 10 females, 10 males), along with clinical evidence of either arrested or delayed puberty, at a mean age of 17.5 years for males and 15.1 years for females. The median latency from tumor diagnosis to HH diagnosis was 35.7 months (IQR 23.2-56.5), but this condition was already present before tumor diagnosis in 2 patients. One 19.2-year-old female patient with suprasellar germinoma manifested secondary amenorrhea 35.1 months after the end of oncologic treatment, probably due to hypothalamic dysfunction and concomitant polycystic ovary syndrome; combined oral hormonal replacement therapy was started with benefit. Finally, no cases of hypergonadotrophic hypogonadism were reported in our cohort.

## Discussion

The clinical history before diagnosis of IC-GCTs can be very variable, ranging from a few months to several years. It may include sudden onset of intracranial hypertension due to third ventricle occlusion and a change in school performance leading to intellectual deficits associated with hypothalamic dysfunction. The neurological signs due to a volume effect on the neuroaxis are hydrocephalus, paralysis of upward gaze, loss of visual acuity or blindness while main endocrinological dysfunction is represented by diabetes insipidus (10, 11). The majority of IC-GCTs occupy suprasellar and/or pineal region. These areas are crucial for the regulation of puberty and hormone production. The hypothalamus-pituitary-gonadal axis is the main neuroendocrine system to regulate reproduction, puberty and sexual hormones production. It works as a cascade: the hypothalamus releases GnRH pulsatily, which stimulates secretion of gonadotropins (LH, FSH) by the pituitary gland in the suprasellar region. This in turn activates gonads (ovaries/testicles) to produce sexual hormones.

The latency period between the onset of GCTs and the development of endocrine complications is highly variable, with many complications emerging within a few months after treatment. Evidence suggests that pubertal dysfunction can manifest even several years to decades following initial cancer therapy, particularly in patients who have undergone radiotherapy or chemotherapy (7). The prevalence of CPP among childhood cancer survivors (CCS) of CNS tumors has been reported to range from 11.9% to 15.2% (12, 13), while gonadotropin deficiency has been observed in 10.8% in the St. Jude cohort of adult CCS, a median of 27.3 years following cranial irradiation (14). Gan et al. reported CPP in 26% and gonadal insufficiency in 20.4% of CCS who had undergone cranial radiotherapy (48–55 Gy) for low-grade gliomas affecting the optic pathway, hypothalamus, and suprasellar region, with a median follow-up of 8.3 years (15). Clement et al. found HH in 4.2% of CCS diagnosed at 12 years or older, with a median follow-up of 6.6 years (16). In our cohort, 45% of subjects (16 males, 13 females) presented with a pubertal disorder, mainly at tumor diagnosis for CPP or PPP and several months after treatment completion for HH. However, 31.7% of patients had a follow-up duration of less than three years and this should be acknowledged as a limitation of the study as pubertal dysfunctions could occur later. For instance, one study reported that 17.4% of patients experienced endocrine dysfunction within this period (17). Long-term endocrine sequelae are also prevalent, with approximately 40% of CCS developing endocrine disorders, many of which arise between one and ten years after therapy (18, 19). While some complications are detected early, others may remain asymptomatic until later in life, highlighting the need for structured, prolonged surveillance and early intervention in CCS to optimize patient outcomes.

The relationship between pubertal status and clinical outcomes in children diagnosed with GCTs is of considerable clinical relevance, particularly in the context of hormonal changes that may influence tumor behavior and treatment response. Emerging evidence suggests that elevated HCG levels play a crucial role in the clinical presentation (i.e. PPP), suggesting a link between pubertal status, tumor type and aggressiveness, and therapeutic strategy. Elevated HCG levels in hormone-secreting GCTs can induce PPP due to its LH-like action, leading to increased testosterone/estradiol production and accelerated physical development in affected children (20, 21). The occurrence of PPP in the context of GCTs has been associated with a more aggressive tumor phenotype, necessitating early diagnosis and intensive therapeutic intervention (21). Children presenting with GCTs and advanced pubertal signs often require individualized therapeutic approaches, incorporating both chemotherapy and surgical management to address the tumor and its endocrine effects (20, 22). Clinical studies indicate that, despite elevated tumor markers and significant pubertal alterations, patients can achieve favorable outcomes with appropriate treatment, including cases of complete remission (22, 23). In our cohort, PPP resolved spontaneously in three patients with tumor cure and decreased HCG levels. Moreover, the case of PPP that later progressed to HH and the cases that progressed to CPP emphasized that the initial presentation (PPP) does not predict the long-term endocrine status. The presence of PP in pediatric patients should prompt a comprehensive evaluation for underlying GCTs, especially in male children, as it may influence treatment decisions and prognostic assessment (21, 24). In our study, 16 subjects were pre-pubertal, while 11 were post-pubertal. To note, two cases displayed HH before tumor diagnosis; since suprasellar GCTs often present with hypothalamic/pituitary dysfunction, this delay in puberty may be a crucial point for early clinical suspicion. The incidence of pubertal dysfunctions in pediatric IC-GCTs has decreased as a consequence of the earlier detection of tumors, which may be attributed to increased awareness and to the widespread use of MRI compared to previous decades (25). It has also been observed that PP in the form of increased testicular androgen production tend to occur more frequently in boys with pineal tumors. Pineal lesions may cause PP either mimicking LH function by circulating hCG or affecting melatonin release and decreasing the inhibition of hypothalamic–pituitary–gonadal axis, although data on the clinical role of melatonin in PP are scarce (21, 26). Males are more likely to be affected by precocious puberty because development of pubertal signs in boys requires LH stimulation alone on Leydig cells, while elevated levels of both FSH and LH are necessary in girls for follicular development and estrogen production (26). Consequently, it is evident that the reappearance of puberty represents the initial indication of tumor reactivation. In patients with a single episode of pubertal rebound, the mean duration of follow-up until recurrence is approximately 10 months (25). Continuous monitoring of hormone levels and tumor markers is essential in these patients, as fluctuations may reflect tumor progression, response to therapy, or recurrence (21). While elevated hormone levels may complicate the clinical picture, they also serve as valuable diagnostic markers, facilitating early detection and timely intervention.

Platinum-based antineoplastic and alkylating agents are frequently used in the treatment of pediatric IC-GCT and may damage the gonads, leading to premature ovarian insufficiency in women and impaired testosterone secretion in men (8). Nevertheless, no cases of hypergonadotrophic hypogonadism due to chemotherapy-related gonadal toxicity were reported in our young cohort, though it is possible that such cases could be detected with longer follow-up. On the contrary, HH was the most frequent pubertal dysfunction in our population, requiring hormonal replacement therapy in all subjects. A timely substitutive therapy in pediatric brain CCS with delayed/arrested puberty is crucial not only for gonadal function but also for psychosocial well-being.

The long-term impact of endocrine complications on the quality of life (QoL) in patients with GCTs is substantial and multifaceted, particularly as these individuals transition into adulthood. Endocrine sequelae, including hormonal deficiencies and metabolic disorders, can contribute to chronic health complications, adversely affecting both physical and psychological well-being. Endocrine disturbances, including pubertal dysfunctions, in GCT survivors are associated with significant impairments in QoL. Compared to their peers, CCS often report diminished sexual function and heightened reproductive concerns (27). Early identification and management of endocrine complications can substantially improve long-term health outcomes, including optimizing growth, body composition, and metabolic health (28). Furthermore, discussions regarding fertility preservation before the initiation of gonadotoxic treatments are crucial for mitigating reproductive challenges (29). Although endocrine complications present significant challenges, some survivors also report an increased appreciation for life and enhanced emotional support, suggesting a complex interplay between health outcomes and overall QoL (27).

## Conclusions

Patients with IC-GCTs are at risk of pubertal dysfunctions, especially those with suprasellar germinomas. Associated pubertal disorders can be heterogeneous, from HCG-driven PPP to CPP or HH, and show a dynamic nature over time, therefore long-term surveillance is recommended. PPP linked to tumor markers might resolve with oncological treatment, unlike CPP which often requires GnRH agonist therapy. This finding supports a watchful waiting approach from an endocrine point of view in specific cases and underlines the link between pubertal dysfunctions and tumor (and tumor markers) evolution, particularly for PPP that could be a clue for tumor recurrence. Therefore, a careful clinical examination of the patients with a focus on pubertal status is recommended, especially in boys. A thorough understanding of the interplay between pubertal status and GCT progression is essential for optimizing treatment strategies and improving patient outcomes and QoL.

## Data Availability

The original contributions presented in the study are included in the article/**Supplementary Material**. Further inquiries can be directed to the corresponding author.

## Glossary

AFP: alpha-fetoprotein
CCS: childhood cancer survivors
CPP: central precocious puberty (gonadotrophin-dependent)
GnRH: gonadotropin-releasing hormone
HCG: β-human chorionic gonadotropin
HH: hypogonadotrophic hypogonadism
IC-GCTs: Intracranial germ cell tumors
MRI: magnetic resonance imaging
PPP: pseudo-precocious puberty (gonadotrophin-independent)
QoL: quality of life
RT: radiotherapy
