# Supplementary material for: Pubertal dysfunctions in intracranial germ cell tumors
Source: Front Endocrinol (Lausanne). 2026 May 19;17:1736152. doi: 10.3389/fendo.2026.1736152 (PMC13226160; doi:10.3389/fendo.2026.1736152)
Supplement: Supplementary file 1 [file Table1.docx]

Supplementary Table 1. Clinical and laboratory data of the 27 cases with pubertal dysfunction

|  | **Age at diagnosis (years)** | **Sex** | **Height (SDS)** | **Pubertal status** | **betaHCG serum (IU/L)** | **betaHCG CSF** | **Surgery** | **Tumour location** | **Metastasis** | **Histology** | **RT (1-YES/0-NO)** | **CT (1=YES/2=NO)** | **Relapses (1=YES/0=NO)** | **Pubertal dysfunction** | **Age at pubertal dysfunction diagnosis (years)** | **Time from Cancer Diagnosis (months)** | **Time from End of Treatment (months)** | **Height (SDS)** | **LH (U/L)** | **FSH (U/L)** | **17 beta estradiol (pmol/L)** | **Testosterone (nmol/L)** | **Treatment** | **Age at alast follow up (years)** | **Time from Cancer Diagnosis (months)** | **Time from End of Treatment (months)** | **Height (SDS)** | **Tanner (PRE/POST pubertal)** | **LH (U/L)** | **FSH (U/L)** | **17 beta estradiol (pmol/L)** | **testosterone (nmol/L)** | **Pubertal dysfunction** | **Growth Hormone Deficiency** | **Adrenal failure** | **Hypothyroidism** | **Death** |
| --- | --- | --- | --- | --- | --- | --- | --- | --- | --- | --- | --- | --- | --- | --- | --- | --- | --- | --- | --- | --- | --- | --- | --- | --- | --- | --- | --- | --- | --- | --- | --- | --- | --- | --- | --- | --- | --- |
| GCT1 | 15,9 | Male | 1,12 | Post-pubertal | 0 | 1 | NO | Bifocal | YES | Germinoma | Proton (CSI+metastasis boost) | YES (PEI) | NO | HH | 15,8 | -1,1 | -4,2 | 1,15 |  |  | / |  | Testosterone injections | 19,1 | 39,1 | 25,0 | **0,9** | Post-pubertal | <0.2 | <0.7 | / | 8.2 | HH | NO | YES | YES | NO |
| GCT3 | 15,3 | Female | 0,23 | Pre-pubertal | 0 | 0 | YES | Suprasellar | NO | Germinoma | VMAT (WVI) | YES (PEI) | NO | HH | 15,9 | 7,3 | 1,4 | 0,13 | LHRH test peak 18 | LHRH test peak 15 | 20 | / | Estradiol patch | 19,3 | 47,5 | 41,7 | **0,58** | Pre-pubertal | 0,6 | 7 | 262 | / | HH | YES | NO | NO | NO |
| GCT5 | 9,1 | Male | 1,01 | Post-pubertal | 3 | 3 | YES | Suprasellar | NO | Teratoma | IMRT (local) | YES (PEI) | NO | PPP | 9,1 | 0,0 | -6,6 | 1,01 | <1 | <1 | / | 42,8 | None | 14,4 | 63,4 | 56,7 | -0,42 | Post-pubertal | <0.2 | <0.2 | / | 2.6 | HH | YES | YES | YES | NO |
| GCT8 | 9,0 | Male | 0,36 | Pre-pubertal | 0 | 1 | YES | Pineal | NO | Germinoma | VMAT (WVI+TB) | YES (PEI) | NO | CPP | 9,8 | 9,3 | 3,7 | -0,34 | LHRH test peak 7 | LHRH test peak 5 | / | <0,35 | Triptorelin 3.75 mg monthly | 14,4 | 64,5 | 58,9 | 0,19 | Post-pubertal | 1 | 1 | / | 14 | None | YES | NO | NO | NO |
| GCT9 | 7,3 | Male | 2,41 | Post-pubertal | 3 | 2 | YES | Pineal | NO | Mixed | VMAT (WVI) | YES (PEI) | NO | PPP -> CPP | 6,8 - 8,06 |  |  | 1,93 | LHRH test peak 0,4 - 5 | LHRH test peak 0,3 - 4,6 | / | >52 - <0,4 | Bicalutamide and Anastrozole daily for 14 months, then Triptorelin 11.25 mg every 10 weeks | 12,0 | 56,1 | 49,2 | 1,75 | Post-pubertal | 0.3 | <0.7 | / | <0.4 | None | YES | NO | NO | NO |
| GCT15 | 17,5 | Male | 0,39 | Post-pubertal | 0 | 0 | YES | Bifocal | YES | Germinoma | VMAT, CyberKnife (CSI + metastasis boost) | YES + PBSC Translpant | YES | HH | 17,9 | 4,1 | 2,5 | 0,28 |  |  | / |  | Testosterone injections | 22,0 | 53,6 | 52,0 | **0,43** | Post-pubertal |  |  | / | 14 | HH | YES | YES | YES | YES |
| GCT16 | 17,3 | Male | -1,03 | Post-pubertal | 0 | 1 | YES | Suprasellar | YES | Germinoma | VMAT (CSI + metastasis boost) | YES (PEI) | NO | HH | 19,3 | 24,7 | 21,3 | -0,65 |  |  | / |  | Testosterone injections | 24,8 | 91,1 | 87,7 | **-0,43** | Post-pubertal |  |  | / |  | HH | YES | YES | YES | NO |
| GCT20 | 10,4 | Male |  | Pre-pubertal | 1 | 2 | YES | Suprasellar | NO | Teratoma | VMAT (WVI+TB) | YES (PEI) | NO | HH | 13,3 | 35,7 | 30,1 | 0,06 |  |  | / |  | Testosterone gel | 17,4 | 84,2 | 78,6 | 0,71 | Post-pubertal | / | / | / | 0,36 | HH | YES | YES | YES | NO |
| GCT21 | 10,6 | Female | -0,06 | Pre-pubertal | 0 | 0 | YES | Suprasellar | NO | Germinoma | VMAT (WVI+TB) | NO | NO | HH | 13,1 | 30,0 | 28,3 | -0,62 | 1 | 2 |  | / | Oral Ethinylestradiol + progesterone | 20,5 | 119,0 | 117,3 | **1,85** | Post-pubertal | / | / | 208 | / | HH | YES | YES | YES | NO |
| GCT26 | 12,3 | Male | -0,64 | Post-pubertal | 2 | 2 | YES | Suprasellar | NO | Teratoma | IMRT (WBI) | YES (PEI) | YES | HH | 14,6 | 26,8 | 20,5 | -2,05 | LHRH test peak <0,2 | LHRH test peak <0,8 | / | <0,9 | Testosterone injections | 23,2 | 130,0 | 123,7 | **-0,73** | Post-pubertal | <0.1 | <0.1 |  | 7,9 | HH | YES | YES | YES | NO |
| GCT28 | 6,8 | Male | 2,21 | Post-pubertal | 0 | 2 | YES | Pineal | NO | Teratoma | IMRT (WBI) | YES (PEI) | NO | PPP | 6,8 | 0,0 | -6,6 | 2,21 | LHRH test peak 2,6 | LHRH test peak 0,7 | / | 16,2 | Triptorelin 3.75 mg 3-weekly,the switch Leuprorelin 11.25mg 8-weekly for poor control of pubertal signs | 19,2 | 149,2 | 142,7 | **0,73** | Post-pubertal | 1,8 | 1,3 | / | 11,2 | None | NO | NO | NO | NO |
| GCT29 | 12,2 | Male |  | Pre-pubertal | 0 | 0 | YES | Pineal | NO | Mixed | VMAT (CSI) | YES (PEI) | YES | HH | 16,8 | 54,5 | 19,1 | -1,59 | 5 | 15 | / | 1,6 | Testosterone injections | 18,5 | 75,5 | 40,0 |  | Post-pubertal |  |  |  |  | HH | YES | NO | NO | YES |
| GCT31 | 9,7 | Female |  | Pre-pubertal | 0 | 0 | YES | Suprasellar | NO | Germinoma | VMAT (WBI) | YES + PBSC Translpant | YES | HH | 14,5 | 58,4 | 51,4 | -2,59 |  |  |  | / | Combined hormonal replacement therapy | 17,9 | 99,2 | 92,2 | **-2,08** | Post-pubertal |  |  |  |  | HH | YES | YES | YES | NO |
| GCT32 | 14,5 | Male | 0,36 | Post-pubertal | 0 | 0 | YES | Pineal | NO | Germinoma | VMAT (CSI) | YES (PEI) | YES | HH | 24,2 | 116,6 | 111,4 | -1,09 | 1 | 3 | / | 12,1 | Testosterone injections | 32,4 | 215,2 | 210,0 | **-1,66** | Post-pubertal |  |  |  |  | HH | NO | NO | NO | NO |
| GCT35 | 16,1 | Male |  | Post-pubertal | 3 | 3 | YES | Pineal | YES | Mixed | VMAT (WBI) | YES + PBSC Translpant | YES | HH | 19,2 | 37,2 | 32,8 |  | 5 | 9 |  | 20,9...3,2 | Testosterone injections | 35,9 | 237,3 | 233,0 |  | Post-pubertal | <0.1 | 0,1 |  | 18.4 (8.6-29) | HH | YES | YES | YES | NO |
| GCT36 | 10,9 | Female |  | Pre-pubertal | 3 | 3 | YES | Suprasellar | NO | Mixed | VMAT (WBI) | YES + PBSC Translpant | YES | HH | 13,3 | 28,7 | 23,0 | -1,01 | LHRH test peak 0,1 | LHRH test peak 0,1 | <30 | / | Oral Ethinyloestradiol | 14,9 | 47,3 | 41,6 |  | Post-pubertal |  |  |  |  | HH | YES | YES | YES | YES |
| GCT37 | 11,3 | Female | -2,49 | Pre-pubertal | 1 | 1 | YES | Suprasellar | NO | Germinoma | VMAT (WVI+WBI) | NO | NO | HH | 12,3 | 12,2 | 9,1 | -2,1 | 0 | 0 |  |  | Oral Ethinylestradiol + progesterone | 29,6 | 219,5 | 216,4 | **-1,48** | Post-pubertal |  |  | 20 | / | HH | YES | YES | YES | NO |
| GCT42 | 16,0 | Female |  | Post-pubertal | 0 | 0 | YES | Suprasellar | NO | Germinoma | VMAT (WVI+WBI) | NO | YES | ?Hypothalamic+PCOS | 19,2 | 37,7 | 35,1 | -0,7 | 1,6 | 3,7 | 70 | / | Combined hormonal replacement therapy | 36,8 | 249,9 | 247,3 | **-0,73** | Post-pubertal | 1,6 | 3,7 | 70 | / | HH | YES | NO | NO | NO |
| GCT43 | 16,5 | Female | -3,53 | Pre-pubertal | 0 | 0 | YES | Suprasellar | NO | Germinoma | VMAT (WVI+WBI) | YES (PEI) | NO | HH | 18,3 | 21,7 | 16,4 | -2,78 | LHRH test peak 3,5 |  | <30 | / | Oral Ethinylestradiol + progesterone | 20,9 | 53,6 | 48,3 | **-2,18** | Post-pubertal | 2 | 7 | 202 (on HRT) | / | HH | YES | NO | NO | NO |
| GCT45 | 7,9 | Female | -0,23 | Pre-pubertal | 0 | 0 | YES | Suprasellar | NO | Germinoma | VMAT (WVI+WBI) | NO | NO | HH | 16,3 | 101,0 | 98,6 | 2,51 |  |  |  | / | Oral Ethinylestradiol + progesterone | 17,2 | 111,4 | 109,0 | **2,49** | Post-pubertal |  |  |  | / | HH | YES | YES | YES | NO |
| GCT46 | 13,6 | Female |  | Pre-pubertal | 0 |  | YES | Suprasellar | NO | Germinoma | VMAT (WVI+WBI) | YES (PEI) | NO | HH |  |  |  |  |  |  |  | / |  | 22,0 | 101,0 | 97,8 | **-0,79** | Post-pubertal |  |  |  |  | HH | YES | YES | YES | NO |
| GCT47 | 10,3 | Male | 0,89 | Pre-pubertal | 0 | 0 | YES | Suprasellar | NO | Germinoma | VMAT (CSI + metastasis boost) | YES + PBSC Translpant | YES | HH | 14,8 | 52,8 | 50,2 | -0,94 |  |  | / |  | Oral Ethinylestradiol + progesterone | 18,7 | 100,7 | 98,0 | **0,3** | Post-pubertal | <1 | <1 | / | 1,7 | HH | YES | YES | YES | NO |
| GCT48 | 6,9 | Female |  | Pre-pubertal | 0 | 0 | YES | Bifocal | NO | Germinoma | VMAT (CSI + metastasis boost) | NO | NO | HH |  |  |  |  |  |  |  | / | Oral Ethinylestradiol + progesterone | 17,9 | 131,4 | 129,5 |  | Post-pubertal |  |  |  | / | HH | YES | YES | YES | NO |
| GCT51 | 5,4 | Female | -2,98 | Pre-pubertal | 0 | 0 | YES | Suprasellar | NO | Germinoma | YES | YES (PEI) | NO | HH | 15,7 | 123,3 | 117,5 | -1,77 | <0.3 | <0.3 |  | / | Oral Ethinylestradiol + progesterone | 18,0 | 151,8 | 146,0 | **-1,42** | Post-pubertal |  |  |  | / | HH | YES | YES | YES | NO |
| GCT52 | 11,1 | Male |  | Pre-pubertal | 0 | 0 | YES | Suprasellar | NO | Germinoma | YES | YES + PBSC Translpant | YES | HH | 23,7 | 150,9 | 146,9 |  |  |  |  |  | None | 29,2 | 216,7 | 212,7 | **0,07** | Post-pubertal |  |  | / |  | HH | YES | YES | YES | NO |
| GCT55 | 10,7 | Male | -0,31 | Pre-pubertal | 1 | 2 | NO | Pineal | YES | Germinoma | YES | YES (PEI) | NO | PPP | 10,8 | 1,3 | -5,4 |  | <0.1 | <0.2 |  | 13.2 | None | 11,8 | 13,3 | 6,6 | -2,3 | Pre-pubertal |  |  |  |  | PPP | NO | YES | YES | NO |
| GCT60 | 10,5 | Male | 0,18 | Post-pubertal | 2 | 3 | NO | Pineal | NO | Germinoma | NO | YES (PEI) | NO | PPP | 10,6 | 0,7 | -0,8 |  | <0,2 | <0.7 |  | 37 | None | 10,6 | 6,7 | -0,8 | 0,18 | Post-pubertal |  |  |  |  | PPP | NO | NO | NO | NO |

*Abbreviations: HH=Hypogonadotrophic hypogonadism; PCOS= polycystic ovary syndrome; PPP= Pseudo-precocious puberty; IC-GCT=intracranial germ cell tumours; CPP= Central precocious puberty; GnRH= gonadotropin-releasing hormone; CSI= craniospinal irradiation; WVI= whole ventricular irradiation; WBI= whole body irradiation; TB= total body; VMAT= Volumetric Modulated Arc Therapy; IMRT= Intensity-modulated radiotherapy; PEI= carboplatinum + etoposide + ifosfamide; PBS= Peripheral blood stem cell*
